# Potential Candidate Genes Associated with Litter Size in Goats: A Review

**DOI:** 10.3390/ani15010082

**Published:** 2025-01-02

**Authors:** Wenting Chen, Ying Han, Yinghui Chen, Xiaotong Liu, Huili Liang, Changfa Wang, Muhammad Zahoor Khan

**Affiliations:** School of Agricultural Science and Engineering, Liaocheng University, Liaocheng 252000, China

**Keywords:** prolificacy, genes, goat reproduction, selective breeding, reproductive performance

## Abstract

This review examines genetic markers associated with litter size in goats, a key reproductive trait impacting productivity in small ruminant farming. Goats play a vital socioeconomic role in both low- and high-income regions; however, their productivity remains limited due to low reproductive efficiency. Litter size, influenced by multiple genes and environmental factors, directly affects farm profitability and sustainability by increasing the output per breeding cycle. Recent advancements in genetic research have identified key genes and pathways associated with reproductive traits, including gonadotropin-releasing hormone (*GnRH*), inhibin (*INHAA*), Kit ligand (*KITLG*), protein phosphatase 3 catalytic subunit alpha (*PPP3CA*), prolactin receptor (*PRLR*), POU domain class 1 transcription factor 1 (*POU1F1*), anti-Müllerian hormone (*AMH*), bone morphogenetic proteins (*BMP*), growth differentiation factor 9 (*GDF9*), and *KISS1* and suppressor of mothers against decapentaplegic (*SMAD*) family genes, among others. These genes regulate crucial physiological processes such as folliculogenesis, hormone synthesis, and ovulation. Genome-wide association studies (GWASs) and transcriptomic analyses have pinpointed specific genes linked to increased litter size, highlighting their potential in selective breeding programs. By incorporating genomic data, breeding strategies can achieve higher selection accuracy, accelerate genetic gains, and improve reproductive efficiency. This review emphasizes the importance of genetic markers in optimizing litter size and promoting sustainable productivity in goat farming.

## Simple Summary

Litter size is a critical reproductive and productive trait in female goats, and it has received considerable attention from both animal breeders and farmers. Enhanced reproductive and productive performance in goats often leads to improvements in the economic and biological efficiency of goat production systems. Over the years, a number of genetic markers associated with litter size have been identified across various goat breeds. This review seeks to provide an in-depth analysis of the identified genetic markers, exploring their potential roles and implications for enhancing litter size in goats.

## 1. Introduction

Small ruminants, including goats, play a vital role in both low-income, food-deficit countries and high-income, technologically advanced nations [1]. In many parts of the world, particularly in regions facing economic challenges, these animals serve as essential sources of food and livelihood. However, despite the socioeconomic value and increasing demand for goat products, global productivity in these species remains low [2]. This limitation is largely due to suboptimal reproductive and productive performance, which directly impacts profitability and sustainability in small ruminant farming.

Enhancing reproductive performance in goats by improving litter size is pivotal for increasing productivity, meeting the demand for meat and dairy, and boosting farm profitability [3]. Selective breeding programs that focus on litter size facilitate genetic improvements, leading to a higher yield of offspring per birth cycle and supporting economic gains by supplying more animals for sale or future breeding. These programs contribute to resource efficiency by reducing production costs and promoting sustainable land use while managing the nutritional and welfare requirements essential for multiple births. Additionally, litter size research contributes to breed-specific reproductive strategies, supporting biodiversity conservation and breed preservation, making it a valuable strategy for sustainable livestock farming.

Since reproductive traits generally exhibit low heritability and respond minimally to phenotypic selection, incorporating genetic information can significantly enhance selection outcomes [4]. Traditional breeding programs for litter size in goats have primarily relied on phenotypic and pedigree data. Due to the low heritability range of litter size (0.08–0.18) [4,5], the rate of genetic improvement is limited. Integrating genomic data into genetic evaluations of prolificacy in goats could therefore increase selection accuracy and accelerate genetic progress.

Litter size is a complex quantitative trait that is controlled by multiple gene [6] loci and environmental factors, as well as maternal influences such as age and intrauterine conditions. Identifying candidate genes associated with economically significant traits and understanding their genomic regions and physiological functions are key to improving breeding outcomes. Methods such as the candidate gene approach, genome-wide association studies (GWASs), and transcriptomic screening are usually utilized to find the causal relationship between genes and reproductive traits, including litter size [4,7,8]. The candidate gene approach quantifies the association between a trait’s phenotypic variation and one or more genes. By using transcriptomic screening, the association of mRNA, miRNA, circular RNA, and long non-coding RNAs with litter size has been well studied [9,10,11,12,13]. Genetic markers linked to reproductive traits enable breeders to enhance selection efficiency in goat breeding programs by allowing for the early identification of animals with desirable genetic traits. These markers facilitate more precise selection for fertility, prolificacy, and overall reproductive health, improving genetic gain and reducing the generation interval [14,15,16,17]. Considering the importance of this topic, this review emphasizes the genetic markers associated with litter size in goats, providing valuable insights that could aid in genomic selection to enhance reproductive efficiency in this crucial livestock species.

## 2. Methodology for Literature Search

This review article synthesizes findings from published studies on genes linked to litter size in goats, covering the period from 2005 to the present. Major academic databases utilized for literature search included Google Scholar, Web of Science, X-MOL, and PubMed. Literature selection followed a targeted keyword strategy, using terms such as “litter size”, “goat”, “genes”, “genetic markers”, “polymorphisms”, “GWAS “, and “genomic selection”. To ensure the rigor and relevance of the data, only studies published in English and indexed in Science Citation Index (SCI) journals were included, while non-English publications and book chapters were excluded to maintain a cohesive and high-quality dataset. Subsequently, genes associated with litter size were analyzed for chromosomal distribution using ShinyGO (ShinyGO 0.81, https://bioinformatics.sdstate.edu/go/ accessed on 26 October 2024) and for biological function and pathway associations through the Database for Annotation, Visualization, and Integrated Discovery (DAVID) bioinformatics tool (https://davidbioinformatics.nih.gov/ accessed on 3 November 2024). These analyses provided enhanced insights into the functional implications of the identified genetic markers.

## 3. Potential Candidate Genes Associated with Litter Size in Goats

**Figure 1:**
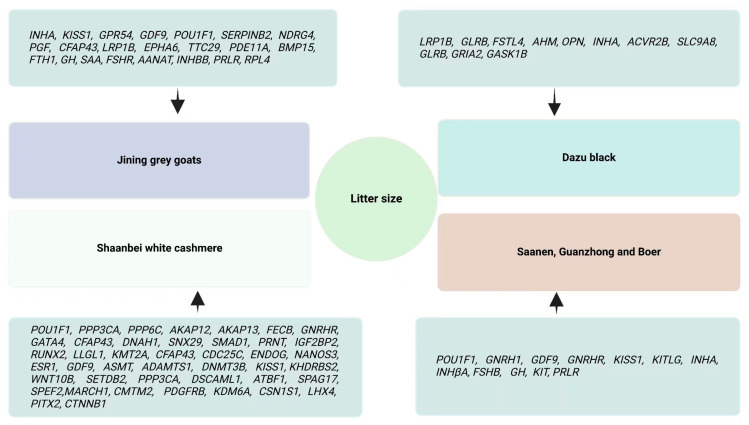
Summary of genes associated with litter size in goats (Jining grey, Guanzhong, Saanen, Boer, Shaanbei white cashmere, and Dazu black goats) reported by previous studies [25,31,34,35,39,42,58,61,67,75,76,84,90,92,93,99,104,105,106,110,123,124,125,126,129,132,152,154,155,156,157,158,159,160,161,162,163,164,165,166,167,168,169,170,171,172,173,174,175,176,177,178,179,180,181,182,183,184,185,186,187,188,189,190,191,192,193,194,195,196,197,198,199,200,201,202].

Litter size in goats, defined as the number of offspring produced per birthing event, is a complex trait regulated by a diverse array of genes involved in reproductive and developmental pathways. These genes contribute to various aspects of reproductive physiology, such as folliculogenesis, hormone regulation, and early embryonic development, all of which are critical determinants of reproductive performance, including litter size. Among the genes investigated, the anti-Müllerian hormone (*AMH*), casein (*CSN*), A-kinase anchoring protein (*AKAP*), bone morphogenetic protein (*BMP*), and suppressor of mothers against decapentaplegic (*SMAD*) gene families have been studied most extensively in relation to litter size in goats. In addition to these gene families, a broader range of individual genes has been associated with litter size based on the current literature. For instance, the growth differentiation factor 9 (*GDF9*) and kisspeptin (*KISS1*) genes have well-documented effects on ovarian function and follicular development. The *KISS1* gene is also involved in the regulation of the hypothalamic-pituitary-gonadal axis. Genes like gonadotropin-releasing hormone (*GnRH*) and its receptor *GnRHR* are pivotal for initiating and regulating the hormonal cascade that leads to ovulation. The follicle-stimulating hormone (*FSH*), growth hormone (*GH*), insulin-like growth factor 1 (*IGF1*), inhibin (*INHAA*), Kit ligand (*KITLG*), protein phosphatase 3 catalytic subunit alpha (*PPP3CA*), prolactin receptor (*PRLR*), and POU domain class 1 transcription factor 1 (*POU1F1*) genes are also consistently reported in the literature for their association with litter size (Table 1 and Figure 1). Additionally, information on the goat breeds, genes reviewed in this study, their chromosomal distribution, and the countries where the studies were conducted are provided in Supplementary Figure S1 and Table S1. These genes influence a variety of pathways related to ovarian follicle development, hormonal response, cellular proliferation, and cellular differentiation, impacting the ovulatory rate and thus contributing to the goat’s reproductive efficiency and litter size potential.

### 3.1. Bone Morphogenetic Protein (BMP) Family Genes and Growth and Differentiation Factor 9 (GDF9) Role in Regulating Litter Size in Goats

The *BMP* family genes and *GDF9* are well-studied members of the transforming growth factor-beta (TGF-β) superfamily and play a crucial role in follicular growth and differentiation, cumulus expansion, and ovulation [18,19,20]. Furthermore, *BMP15* and *GDF9* are oocyte-secreted factors with a leading role in the control of ovarian function in female reproduction, modulating both the cell fate of the somatic granulosa cells and the quality and developmental competence of the egg [20,21,22,23]. Consistently, a recent study reported that *GDF9* and BMP receptors (*BMPRs*) enhance the proliferation of granulosa and theca cells [24]. Studies have consistently demonstrated the pivotal role of *BMP* family genes, particularly *BMP4* [20,25], *BMP15* [14,26,27,28,29,30], and *BMPR1B* (also known as *FECB*) [11,31,32,33], in influencing litter size in goats. The lambing rate for individuals with the CC and CT genotypes at the *FecB* C94T locus was significantly higher compared to those with the TT genotype, with increases of 45.7% and 46.8%, respectively. Additionally, the lambing rate for individuals with the CC genotype at the *ESR* C463T locus was significantly greater than that observed in both CT and TT genotypic individuals, showing an increase of 9% and 15%, respectively [31]. Wang et al. [30] conducted a study utilizing polymerase chain reaction–single-strand conformation polymorphism (PCR-SSCP) and DNA sequencing to analyze exon 2 of the *BMP15* gene in two local goat breeds in China. Their findings indicated that the Funiu white goats with the BB genotype had a significantly higher litter size at birth, with an average of 0.91 or 0.82 more kids compared to those with the AB or AA genotypes, respectively. Additionally, they highlighted that litter size during the second kidding is often considered a critical indicator of a goat’s prolificacy [30]. The ability of a goat to consistently produce multiple offspring in consecutive pregnancies serves as a key measure of its reproductive health. A larger litter size during the second kidding not only suggests the goat’s fertility but also its capacity to conceive and successfully carry multiple fetuses. Moreover, the number of offspring produced, particularly in successive births, is often reflective of the genetic traits inherited from both the doe and the sire, with goats showing higher productivity potentially carrying genetic markers associated with enhanced fertility and prolificacy. Consequently, the polymorphisms (g.3548A>G and g.3699G>A) in *GNRH1* and *GDF9* (g.4093G>A) had significant effects on litter size and could serve as genetic markers for litter size in goat breeding [34]. Consistently, another study found the association of SNPs (g.3905A>C and g.4135G>A) with litter size in Shaanbei white cashmere goats [35]. Other studies also provided significant data on the association of polymorphisms in the *GDF9* gene with litter size in different breeds of goats [26,36,37,38,39]. Based on the above discussion, it can be concluded that *BMP* family genes and *GDF9* demonstrate a strong association with litter size in goats, largely due to their roles in ovarian follicle development, ovulation regulation, and the modulation of follicular response to reproductive signals, highlighting their potential as valuable genetic markers in goat breeding programs aimed at increasing litter size.

### 3.2. The AMH, SMAD, and INH Genes’ Association with Litter Size in Goats

The anti-Müllerian hormone (*AMH*), growth differentiation factor 9 (*GDF9*), sterile alpha motif domain-containing (*SAMD*), and inhibin (*INH*) genes are crucial members of the transforming growth factor-beta (TGF-β) superfamily, which is broadly known for its roles in reproductive physiology, cellular differentiation, and tissue growth [40]. These genes have been shown to be significantly associated with litter size in goats, making them targets of interest for genetic improvement in goat breeding programs aimed at enhancing reproductive output [41]. Furthermore, studies have reported that an SNP-g.89172108A>C within the *AMH* gene was significantly associated with litter size in Chuanzhong black and Dazu black goats [41,42]. Furthermore, they reported that Chuanzhong black goats with the TT genotype exhibited a higher litter size compared to those with the TG and GG genotypes [41]. Meanwhile, Han et al. found that the litter size in goats with the homozygous CC genotype was significantly higher compared to those with the heterozygous AC genotype, suggesting that this SNP may represent a beneficial mutation associated with enhanced fertility in goats. Furthermore, the second-born litter size was greater in goats with the CC genotype than in those with the AA genotype [42]. Interestingly, a study reported that serum AMH concentration was associated with ovarian reserve, antral follicle count, ovarian surface area, ovulation rate, and litter size in goats [43,44] and cattle [45]. The *AMH* gene has also been found to be associated with litter size in Romanov sheep [46]. In addition, a study found several key genes, including *SMAD2* and *AMHR2*, that were involved in litter size in goats [47].

The SMAD protein family, encompassing the *SMAD1*, *SMAD2*, *SMAD3*, *SMAD4*, and *SMAD5* genes, plays a pivotal role in ovarian cell stimulation and regulation, as supported by findings from several studies [48,49,50,51]. Notably, GWASs have identified *SMAD1* as a significant contributor to litter size in sheep, with high-prolificacy sheep exhibiting higher expression of this gene [52]. Furthermore, SMAD proteins function as intracellular signaling mediators within the transforming growth factor-beta (TGF-β) superfamily, displaying widespread expression across both developmental and adult tissues, with a pronounced role in normal embryogenesis [48,53,54]. Ovary-specific interactions between SMAD proteins and other regulatory molecules, such as transcription factors and co-modulators, further suggest a critical function in directing TGF-superfamily ligand responses towards specific gene targets within ovarian tissues [53]. Additionally, the *SMAD1* gene activates a key BMP intracellular signal transduction pathway that has been associated with accelerated ovulation in ewes [52,54,55]. Through its encoding of SMAD proteins that drive BMP signaling, *SMAD1* has been implicated in amplifying the actions of key reproductive candidate genes [55]. In addition, they noticed the effects of the Booroola (*FecB*) gene on progesterone levels and the expression of BMP and SMAD signaling genes in the ovaries of ewes [55]. Emerging evidence also indicates that *SMAD1* is expressed in ovarian tissues, contributing to the regulation of follicular growth and ovulation in livestock through pathways involving estrogen, TGF-β, retrograde endocannabinoid signaling, and the Hippo pathway [52,56]. Similarly, SMAD signaling in granulosa cells of mice, for example, has been suggested to play a role in regulating metastatic behaviors or other ovarian functions [57]. The *SMAD2* gene, similarly, has shown a significant association with litter size and has been documented in goat ovarian tissue, reinforcing its reproductive relevance [58]. Moreover, studies consistently underscore the association between the expression and polymorphisms of *SMAD1*, *SMAD2*, *SMAD3*, and *SMAD6* and litter size in goats, highlighting a critical genetic component influencing reproductive traits in livestock [58,59,60,61].

### 3.3. Inhibins and Their Role in Regulating Litter Size in Goats

Inhibins, specifically inhibin-A (INH-A) and inhibin-B (INH-B), are glycoproteins primarily secreted by ovarian granulosa cells, playing a critical role in the endocrine regulation of reproduction. These proteins function as feedback inhibitors of follicle-stimulating hormone (FSH) in the anterior pituitary, a mechanism crucial for modulating follicular development and ovulation [62,63]. Elevated inhibin levels modulate FSH concentrations, thereby influencing ovulation rates and follicular recruitment, factors closely associated with increased litter size in goats [64,65]. Studies have revealed that genetic variants in INH genes correlate with enhanced litter size, with specific alleles being more frequent in high-fertility goat populations [66,67,68,69,70,71]. The litter size of individuals with the AC genotype at the g.28317663A>C locus of the *INHA* gene was significantly higher than that of individuals with the AA genotype. This SNP, which alters the amino acid sequence, may influence the function of the INHA protein by affecting its structure [66]. Consistently, Isa et al. reported that the CT genotype at the g.3234C>T locus in the *INHA* gene was associated with a significantly larger litter size compared to the CC genotype in West African Dwarf goats [69].

### 3.4. Gonadotropin-Releasing Hormone Receptor (GnRHR) Gene and Its Role in Litter Size

The gonadotropin-releasing hormone receptor (*GnRHR*) gene is a prominent candidate gene for litter size due to its pivotal role in the hypothalamic-pituitary-gonadal (HPG) axis, which regulates the synthesis and release of gonadotropins [72,73]. The *GnRHR* binds with high affinity to the gonadotropin-releasing hormone (*GnRH*) on pituitary gonadotropes, stimulating the secretion of luteinizing hormone (LH) and FSH. This interaction is essential for regulating ovarian function, including hormone synthesis and gamete production [74,75]. Enhanced *GnRH* secretion and increased pituitary *GnRHR* concentrations facilitate ovulation in mammals, whereas the absence of *GnRHR* may inhibit ovulation, potentially leading to infertility [74]. Numerous studies indicate an association between polymorphisms in *GnRH* and its receptor *GnRHR* with increased litter size in goats [72,76,77,78,79,80]. Furthermore, a significant association was observed between the GG genotype of g.-29T>G in the *GnRHR* gene and an increased mean litter size in goats when compared to the GT and TT genotypes [73]. In addition, they observed that the litter size significantly increased from the first to the fourth parity, which revealed the genetic potential of West African Dwarf goats [73]. Furthermore, individuals with the AA genotype of the GnRHR gene exhibited a significantly larger litter size compared to those with the AC genotype, from the first to the third parity, in both Shaanan and Boer goats [76].

### 3.5. Regulation of Reproductive Function by KISS1/GPR54 Signaling Pathway

The KISS1/GPR54 signaling pathway is essential for initiating GnRH secretion, which, in turn, regulates gonadotropin release [81,82,83]. In females, this signaling pathway operates through feedback loops to induce the pre-ovulatory LH surge, playing a significant role in reproductive control [84]. Given its integral role in reproductive physiology, *KISS1* is critical for fertility monitoring and regulation [85,86]. Consistent with this, studies have demonstrated that the *KISS1* gene substantially contributes to multiparity in goats [84,87]. Moreover, *KISS1* gene polymorphisms have been linked to various reproductive traits, including litter size, with several studies confirming the gene’s significance in reproductive efficiency [88,89,90,91,92,93]. It has been further documented that the GG genotype at the g.893C>G locus of the *KISS1* gene is associated with an increased litter size across three parities, compared to the CC and GC genotypes, in both Iraqi and Cypriot goat populations [89]. Similarly, individuals with the AA genotype had greater litter size than those with GG at the g.1384G>A locus in Guanzhong goats [90]. The G-protein-coupled receptor (GPR54) serves as the specific receptor for a group of neuropeptides known as kisspeptins, which are derived from the *KISS1* gene. The KISS1/GPR54 system is recognized as a crucial regulator of the onset of puberty in mammals [94]. Thus, polymorphisms in the G-protein-coupled receptor *(GPR54)* might have a significant effect on goat reproductive traits. Consistently, several studies have previously explored the influence of polymorphisms such as C1122T in exon 1, T1830C, g.2124T>A, and g.2270C>T in *GPR54* on litter size across various goat breeds [94,95,96,97,98,99,100].

### 3.6. Role of KITLG, AKAP, and PPP Family Genes in Goat Litter Size

The KIT ligand (*KITLG*) and its receptor *KIT* are integral to the migration, proliferation, and survival of primordial germ cells (PGCs), underscoring their importance in reproductive biology [101]. Research consistently reports associations between *KITLG* gene polymorphisms and reproductive performance, including litter size, in goats [102,103,104,105,106]. Additionally, miR-9, a microRNA involved in regulating *KITLG* and *IGF1*, has been shown to impact reproductive efficiency in goats. Polymorphisms in *miR-9* may therefore influence reproductive outcomes [107].

The association between A-kinase anchoring proteins (*AKAPs*) and litter size has been previously established. *AKAPs* are key mediators in localizing protein kinase A (PKA) within the cell, modulating the specificity and kinetics of substrate phosphorylation. Research has shown that interactions between *AKAPs* and PKA promote oocyte maturation even in the sustained presence of high cAMP levels [108,109]. Specifically, *AKAP13* has been associated with estrogen receptor (ER) and progesterone receptor (PR) activities, demonstrating a significant effect on litter size in goats [110]. Additionally, *AKAP12* has been implicated in the function of ovarian granulosa cells in mammals [111]. In addition, Mahmoudi et al. [112] also reported a significant association between the *GABRA5* and *AKAP13* genes and litter size in goats, as identified through GWASs. Recent studies have further identified structural variations in the *AKAP12* gene that correlate with increased litter size in goats [113].

Furthermore, serine/threonine phosphoprotein phosphatases (*PPPs*) represent the primary class of protein phosphatases involved in the regulation of oocyte meiosis and spermatogenesis [114,115,116]. Recent findings have indicated that the *PPP6C* gene is associated with litter size in goats [117]. Correspondingly, studies have found that copy number variations in *PPP3CA* are correlated with litter size in goats [118,119,120], while the *PPP2R5C* gene has also been linked to litter size in goats [121]. This growing body of research underscores the role of *AKAP* and *PPP* family genes in reproductive traits and suggests that they may serve as valuable genetic markers for breeding strategies aimed at optimizing litter size in goats.

### 3.7. POU (Pit-Oct-Unc) Class 1 Homeobox 1 Gene (POU1F1) Association with Litter Size in Goats

The POU (Pit-Oct-Unc) class 1 homeobox 1 gene (*POU1F1,* also known as *Pit-1*) is a critical transcription factor that directly regulates genes associated with pituitary hormones, specifically growth hormone (*GH*) and prolactin (*PRL*). This gene has been identified as a candidate for influencing reproductive and growth traits in goat breeding programs [122]. Several single-nucleotide polymorphisms (SNPs), such as c.682G>T, c.837T>C, and g.34236169A>C, within the *POU1F1* gene have demonstrated associations with litter size across various goat breeds [123,124,125,126]. Additionally, research has consistently highlighted the significant role of the *GH* and *PRLR* genes in reproductive traits, particularly litter size [127,128]. The *GH* gene, in particular, has been implicated in superovulation and follicular development processes [129]. Studies also indicate that PRLR has a substantial effect on litter size in goats [130,131,132]. Additionally, studies in sheep have identified associations between genetic variants in both the *GHR* and *PRLR* genes and variations in litter size [127].

**Table 1:** Summary of studies reporting genes associated with litter size in goats.

Genes	Goat Breed	References
*LRRTM4*	Youzhou dark goats	[7]
*BMP4*	Indian goats	[20]
*BMP15*, *BMPR1B*, *GDF9*	Black Bengal goats	[26,29,33]
*GDF9*	Goats	[37]
*AHM*	Chuanzhong black and Dazu black goats	[41,42]
*CCNB2*, *DNMT3B*, *SMAD2*, *AMHR2*, *FGFR1*, *KDM6A*	Laoshan dairy goats	[47]
*SMAD2*	Goats	[59,60]
*INHA*	Jining Grey, Hainan black, Liaoning Cashmere, Wendeng, Taihang goats, Kalahari Red, West African Dwarf, and Red Sokoto	[66,68,69]
*GnRHR*	West African Dwarf, Shaanan, Boer, and Malabar goats	[72,73,76,77]
*KISS1*	Guanzhong, Saanen, Boer black, Jining Grey Barbari, Beetal, Sirohi, Sojat, Cypriot, and Iraqi goats	[88,89,90,92,93]
*MIR9*, *GABRA5*, *AKAP13*	Markhoz goats	[107,112]
*AKAP12*	Shaanbei white cashmere goats	[113]
*PPP6C*	Shaanbei white cashmere goats	[117]
*PPP3CA*	Shaanbei white cashmere goats	[118]
*GH*	Boer and Matou	[129]
*PRLR*, *IGF1*, *LEP*	Egyptian Zaraibi goats	[130]
*PRLR*, *LHβ*	Boer goats	[132]
*BLM*	Guizhou white goats	[133]
*ACSS2*, *HECW2*, *KDR*, *LHCGR*, *NAMPT*, *PTGFR*, *TFPI*	Goats	[134]
*ITGAV*, *LRP4*, *CDH23*, *TPRN*, *RYR2*, *CELSR1*	Beichuan white goats	[135]
*BMP15*, *BMPR1B*	Kacang and Boerka goats	[136]
*GHRL*	Malabari and Attappady black goats	[137]
*OLR1*	Guizhou white goats	[138]
*CSN3*, *TCF4*	Hechuan white goat, Banjiao goat, and Youzhou dark goats	[139]
*CTSS*	Qianbei Ma goats	[140]
*PPP2R5C*, *SLC39A5*	Yunshang black goats	[141]
*KITLG*, *KISS1*, *GHR*	Nubian goats	[102]
*CTSD*	Qianbei Ma	[142]
*INHA*	Malabari goats	[143]
*PRP1*, *PRP6*	Laoshan dairy goats	[144]
*SIRT3*	Malabari and Attappady Black goats	[145]
*GnRHR*	Non-descript local Sri Lankan goats	[146]
*KISS1*	Damascus and Zaribi goats	[147]
*NGF*	Malabari and Attappady Black goats	[148,149]
*PRLR*	Boer and Macheng Black goats	[150]
*BMPR1B*, *GDF9*, *BMP15*, *FSH, FSHR*, *POU1F1*, *PRLR*, *KISS1*, *GPR54*, *GH*, *INH*, *CART*, *GnRH*, *GnRHR*, *LH*, *BMP4*, *KITLG*, *MT2*, *CYP21*, *AANAT*	Goats	[151]
*FER1L4*, *SRD5A2*	Anhui white goats	[152]
*BMP15*	Alpine, Zaraibi, Baladi, Funiu white, and Taihang black goats	[27,30,153],
*FSHB*	Boer and Matou black goats	[154]

## 4. Gene Ontology (GO) and KEGG Enrichment Analyses of Goat Litter Size-Linked Genes and Their Association with Reproductive Functions

**Figure 2:**
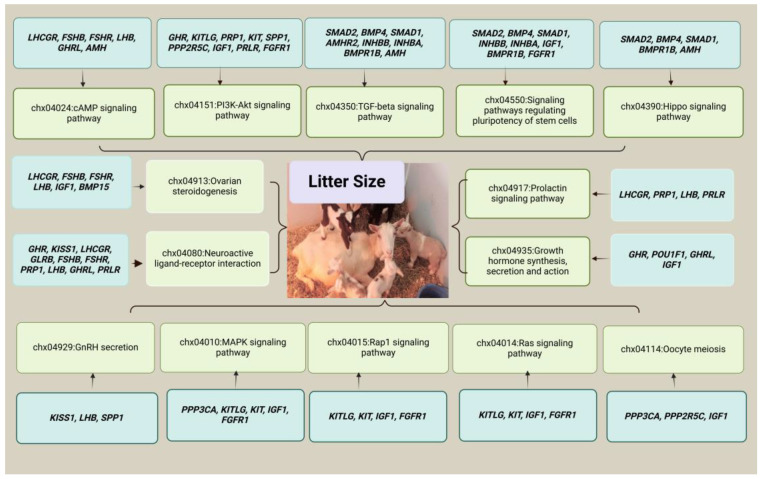
Kyoto Encyclopedia of Genes and Genomes (KEGG) signaling pathways linked to reproductive performance and litter size in goats. Here, it is important to note that this figure is based on speculative information rather than confirmed data, so the relationships shown should be interpreted carefully.

**Figure 3:**
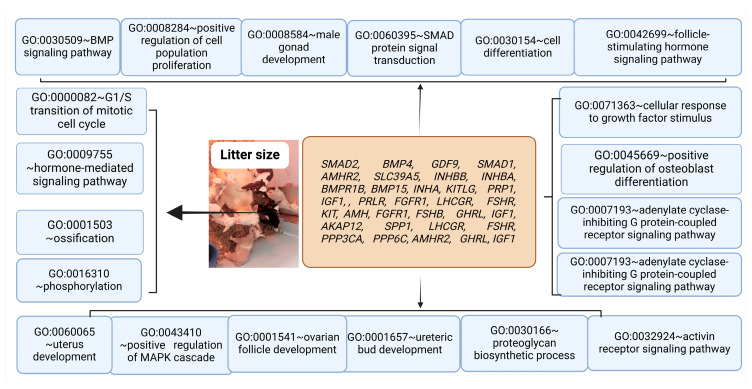
Biological functional processes linked to reproductive performance and litter size. Figure 3 illustrates the biological processes associated with genes influencing litter size. Key functional processes identified include suppressor of mothers against decapentaplegic (SMAD) protein signal transduction, cell differentiation, bone morphogenetic proteins (BMP) signaling pathway, follicular development, and the transition of the mitotic cell cycle, among others.

The genes from Table 1 and Figure 1 were uploaded to the DAVID database for Gene Ontology and Kyoto Encyclopedia of Genes and Genomes (KEGG) enrichment analyses [203]. Furthermore, we also used ShinoyGO [204] for the chromosomal distribution of genes from Table 1 and Figure 1. Based on KEGG and GO analysis, we found that these genes were involved in the regulation of several key signaling pathways, including the Hippo signaling pathway, the TGF-β signaling pathway, ovarian steroidogenesis, GnRH secretion, oocyte meiosis, the cAMP signaling pathway, the prolactin signaling pathway, the PI3K-Akt signaling pathway, the BMP signaling pathway, SMAD protein signal transduction, the follicle-stimulating hormone signaling pathway, cell differentiation, and positive regulation of cell population proliferation, as shown in Figure 2 and Figure 3. The Hippo signaling pathway is crucial in regulating the early stages of mammalian development and has been shown to play a pivotal role in various biological processes. In mammalian preimplantation embryos, this pathway orchestrates cellular events critical to embryo viability and developmental potential [205,206,207]. The pathway’s influence extends into embryogenesis and organogenesis, where it helps shape tissue formation, guiding cells in organizing into functional structures and forming major organ systems [208,209]. Additionally, within the ovarian follicle, the Hippo pathway supports follicular growth and activation, which are necessary for successful ovulation and fertility, as observed in recent studies [210]. Beyond follicle dynamics, it also influences steroidogenesis—the process by which steroids like estrogen and progesterone are produced—ensuring that hormonal balance supports reproductive and systemic health. These diverse functions are mediated through the pathway’s regulation of key biological mechanisms, including cell proliferation, migration, differentiation, and cell fate determination, which allow cells to respond precisely to developmental cues and maintain tissue integrity [211]. The TGF-β pathway is initiated upon the binding of a TGF-β ligand—such as TGF-β itself, activins, BMPs, or GDFs—to a receptor complex located on the surface of target cells. These receptors, classified as serine/threonine kinase receptors, activate a phosphorylation cascade upon ligand binding. This cascade subsequently activates intracellular proteins known as SMADs, which function as signaling molecules that translocate to the nucleus to regulate gene expression. Through the modulation of gene expression, the TGF-β pathway regulates the process of granulosa cell steroidogenesis and apoptosis [212,213] and reproductive performance [214,215]. Other key signaling pathways, including the PI3K-Akt, prolactin, ovarian steroidogenesis, and GnRH signaling pathways, which are associated with litter size in goats, were also regulated by genes linked to this trait [9,11,216]. It has been further confirmed that the neurotrophin receptor B (*NTRK2*) gene, part of the neurotrophic tyrosine receptor kinase family, plays a crucial role in mammalian reproduction, particularly in gamete production and quality. In sheep granulosa cells, overexpression of *NTRK2* enhances cell proliferation and steroid synthesis and follicular development via the PI3K-AKT signaling pathway [216]. 

## 5. Conclusions

Altogether, we provided a comprehensive overview of genes and their possible association with litter size in goats. The reviewed studies emphasize that litter size is a complex quantitative trait influenced by multiple genes and their related cell signaling pathways as well as environmental factors. Key gene families, such as *BMP*, *SMAD*, *AMH*, and *GDF9*, along with regulatory factors like *GnRH* and *KISS1*, are significantly associated with litter size, each playing important roles in reproductive signaling, follicular development, and ovulation processes. Identifying polymorphisms within these genes allows for more precise selection strategies in goat breeding programs, moving beyond traditional phenotypic selection to more effective genomic approaches. By integrating genomic data, breeders can target specific markers to enhance prolificacy, thereby supporting food security and economic stability, particularly in regions where goats play a vital role in subsistence farming. The findings presented in this review provide a foundational basis for further advancements in marker-assisted selection to optimize reproductive performance in goats. However, this review has certain limitations, including the lack of consideration of sample sizes, more comprehensive genotypic data, and a deeper exploration of parity effects. In addition, future studies should consider the heritability of genes associated with litter size across various goat breeds.

## Data Availability

Data sharing is not applicable. No new data were created or analyzed in this study.

## Supplementary Materials

The following supporting information can be downloaded at www.mdpi.com/article/10.3390/ani15010082/s1, Figure S1: Chromosomal distribution; Table S1: Information of genes, breed and region.
