# Supplementary material for: Potential Candidate Genes Associated with Litter Size in Goats: A Review
Source: Animals (Basel). 2025 Jan 2;15(1):82. doi: 10.3390/ani15010082 (PMC11718837; doi:10.3390/ani15010082)
Supplement: Supplementary file 1 [file animals-15-00082-s001.zip › Supplementary Table S1-Animals.pdf]

Supplementary Table S1: Information of genes, breed and region

Genes	Polymorphism/location/amino acid change	Reference sequence/ position referenced/Accession No	Goat breed	Region
AANAT	C586T/ Arg96Trp	NM_001009461	Jining Grey	China
ACSS2, HECW2, KDR, LHCGR, NAMPT, PTGFR, TFPI			Goat	China
AHM	g.89172108A > C (Exon 4)	XM_018050765.1	Dazu black goat	China
AKAP12			Shaanbei white cashmere goats	China
AKAP13			Shaanbei white cashmere goats	China
AMH	g.89172108T >G		Chuanzhong black Goat	China
ASMT<br>*ADAMTS1*	g.158122T>C<br>g.7979477C>T	NW_017189541.1<br>NC_030808.1	Shaanbei white cashmere goats	China
ATBF1			Shaanbei white cashmere goats	China
BLM	g.65796 C > T/ exon 15<br>g.89621 T > G/ exon 22		Guizhou white goats	China
BMP15	C735A （exon 2）<br>G754T （exon 2）<br>C781A （exon 2）	NM_001285588.	Black Bengal goat	Bangladesh
BMP15			Alpine, Zaraibi, and Baladi,	Egypt
BMP15			Funiu white and Taihang black goats	China
BMP15	c.963A>G		Goats	Iran
BMP15, BMPR1B			Kacang and Boerka goat	Indonesia
BMP4			Indian goats	India
BMP4			Jining Grey goats	China
BMPR1B, BMP15, GDF9			Black Bengal goats	India
BMPR1B, GDF9, BMP15, FSH, FSHR, POU1F1, PRLR, KISS1, GPR54, GH, INH, CART, GNRH, GNRHR, LH,			Goat	India

BMP4, KITLG, MT2, CYP21, AANAT				
CCNB2, DNMT3B, SMAD2, AMHR2, FGFR1, KDM6A,			Laoshan dairy goat	China
CDC25C, ENDOG, NANOS3			Shaanbei white cashmere goat	China
CFAP43			Shaanbei white cashmere goats	China
CFAP43			Shaanbei white cashmere goats	China
CFAP43			Shaanbei white cashmere goats	China
CMTM2			Shaanbei white cashmere	China
CSN1S1			Shaanbei white cashmere	China
CSN3, TCF4			Hechuan white goat, Banjiao goat, and Youzhou dark goat	China
CTNNB1			Shaanbei white cashmere	China
CTSD			Qianbei Ma	China
CTSS	g.7413C >T(p.S161F)<br>g.8816A >T (p.N219Y)	NC_030810.1	Qianbei Ma goats	China
DNAH1			Shaanbei white cashmere goats	China
DNMT3B			Shaanbei white cashmere goats	China
DSCAML1			Shaanbei white cashmere goats	China
FECB			Shaanbei white cashmere	China
FECB			Shaanbei white cashmere	China
FECB<br>*ESR1*	C94T<br>C463T		Shaanbei white cashmere goats	China
FER1L4, SRD5A2			Anhui white goats	China
FGF2			Busheri, Saanen and Boer goats	Iran
FSHB			Boer and Matou black goat	China
FSHR			Jining Grey goats	China
FSTL4			Dazu black goat	China
FTH1, GH, SAA			Jining Grey goats	China
GABRA5<br>*AKAP13*	rs268288690<br>rs268256209		Markhoz Goat	Iran
GATA4			Shaanbei white cashmere	China
GDF9	c.1189G>A (p. Gly397Arg)	NM_000143.4	Meta-analysis	Iran

GDF9			Jining Grey goats	China
GDF9	A959C/exon 2/ Gln320Pro	AF078545	Jining Grey goats	China
GDF9<br>*BMP15*	g.233C>A/ exon I<br>g.755T>G/ exon II	EU883989.1<br>EU743938.1	Markhoz goats	Iran
GDF9			Saanen goats	China
GDF9	g.3905A > C/ p.Gln320Pro	NC_022299.1	Shaanbei white cashmere	China
GDF9			Shaanbei white cashmere goats	China
GDF9, BMP15, BMPR1B			Black Bengal	India
GH			Boer and Matou	China
GHRL			Malabari and Attappady black goat	India
GNRH1<br>*GDF9*	g.3548A>G<br>g.4093G>A/(p.Val397Ile)	JN645280<br>JN655693	Xinong Saanen, Guanzhong and Boer	China
GnRHR	c.–29 T>G<br>c.48 G>A<br>c.75 G>A<br>c.209 T>G<br>c.–33A>G	rs644967845<br>rs670739841<br>rs644305875<br>rs640973604	Malabari goat	India
GnRHR	g.-29T > G/ 5'UTR	rs644967845/ NC_022298.1	West African Dwarf	Nigeria
GnRHR			Shaanan and Boer goats	China
GnRHR	G891T<br>G757A		Boer goats	China
GnRHR	G757A/ exon 1<br>G891T/ exon 1	L42937.1	Non-descript local Sri Lankan goats	Sri Lanka
GPR54	C4152T/Exon 1		Jining Grey goats	China
IGF2BP2			Shaanbei white cashmere goats	China
INHA	g.28317663A>C/Exon 2/ Thr→Pro	NC_030809.1	Hainan black goats	China
INHA	G841A/Exon 1/	EF602161.1	Jining Grey, Liaoning Cashmere, Wendeng Dairy and Taihang goats.	China

INHA	g.3234 C> T/ 3'UTR	rs670889338	Kalahari Red, West African Dwarf and Red Sokoto	Nigeria
INHA			Malabari goats	India
INHA, ACVR2B			Dazu black goats	China
INHA, INHβA			Xinong Saanen, Guanzhong and Boer	China
INHBB			Jining Grey goats	China
ITGAV<br>*TPRN*<br>*CELSR1*<br>*LRP4, CDH23, RYR2,*	c.38C > T<br>c.133A > T, c.1192A > G, c.1250A > C<br>c.7640T > C		Beichuan white goat	China
KDM6A			Shaanbei white cashmere	China
KISS1	g.2540C>T	GU142847	Barbari, Beetal, Sirohi and Sojat goats	India
KISS1	g893C>G/exon 1	KC989928.1	Cyprus and Iraqi black goats	Iraq
KISS1	g.1384G>A	JX047312	Guanzhong and Boer goats	China
KISS1	T2643C		Xinong Saanen, Guanzhong and Boer	China
KISS1	G296C/ exon 3	GU142847	Jining Grey goats	China
KISS1			Damascus and Zaribi goat	Egypt
KISS1			Xinong Saanen, Guanzhong and Boer	China
KISS1, KHDRBS2, WNT10B, SETDB2, PPP3CA			Shaanbei white cashmere goats	China
KIT	g.88430T > A<br>g.120466G >A	KF364483	Xinong Saanen, Guanzhong and Boer goat	China
KITLG	g.12654G>A<br>g.12772G>A<br>g.12829T>C<br>g.13090G>T	KM609289	Xinong Saanen and Guanzhong goat	China

	g.13664C>A			
KITLG	g.769T>C<br>g.817G>T<br>g.9760G>C		Xinong Saanen, Guanzhong and Boer	China
KITLG<br>*KISS1*<br>*GHR*	g.18047318 G>A<br>g.1341674 C>G<br>g.32134187G>A	NC_030812.1<br>NC_030823.1<br>NC_030827.1	Nubian goat	China
KITLG	c.1389C > T/ 3′-UTR<br>c.1457A > C/3′-UTR<br>c.1520G > A/3′-UTR	KR869087	Guanzhong dairy goats	China
KMT2A			Shaanbei white cashmere goats	China
LHX4, PITX2			Shaanbei white cashmere	China
LLGL1			Shaanbei white cashmere goats	China
LRP1B, GLRB			Dazu black goats	China
LRRTM4			Youzhou Dark Goats	China
MARCH1			Shaanbei white cashmere	China
MIR9			Markhoz goats	Iran
NGF	g.217G>A/ exon 3<br>g.705G > A/ exon 3	KF724722	Malabari and Attappady Black goats	India
OLR1	g.294 T > A<br>g.2260 T > C<br>g.2268 C > T		Guizhou white goats	China
OPN (SPP1)			Dazu Black, Lezhi Black, Hexi Cashmere	China
PDGFRB			Shaanbei white cashmere	China
POU1F1			Saanen goats	Turkey
POU1F1	c.682G > T<br>c.837T > C	NC_030808.1	Shaanbei white cashmere goats	China
POU1F1			Shaanbei white cashmere	China
POU1F1			Jining Grey goats	China
PPP2R5C<br>*SLC39A5*	g.65977743C>T<br>g.50676693T>C		Yunshang black goats	China
PPP3CA			Shaanbei white cashmere goats	China

PPP6C			Shaanbei white cashmere goats	China
PRLR	g.185275C/T	JX087440.1	Boer and Macheng Black goat	China
PRLR	c.1457G>A/exon 9/p. Ser485Asn<br>c.1645G>A /exon 9/p. Val548Met		Guanzhong and Boer	China
PRLR			Jining Grey goats	China
PRLR and *LHβ*			Boer goats	China
PRLR, IGF1, LEP			Egyptian Zaraibi goat	Egypt
PRNT	c.71A>G	AM412782.1	Shaanbei white cashmere goats	China
PRP1, PRP6			Laoshan dairy goats	China
RPL4			Jining grey goats	China
RUNX2			Shaanbei white cashmere goats	China
SIRT3	c.691C > T/ exon 5	MF176159	Malabari and Attappady Black goats	India
SLC9A8, GLRB, GRIA2, GASK1B			Dazu black goat	China
SMAD1			Shaanbei white cashmere goats	China
SMAD2			Goat	China
SNX29			Shaanbei white cashmere	China
SPAG17			Shaanbei white cashmere goats	China
SPEF2			Shaanbei white cashmere	China
