# Supplementary figures and images for: Potential Candidate Genes Associated with Litter Size in Goats: A Review
Source: Animals (Basel). 2025 Jan 2;15(1):82. doi: 10.3390/ani15010082 (PMC11718837; doi:10.3390/ani15010082)

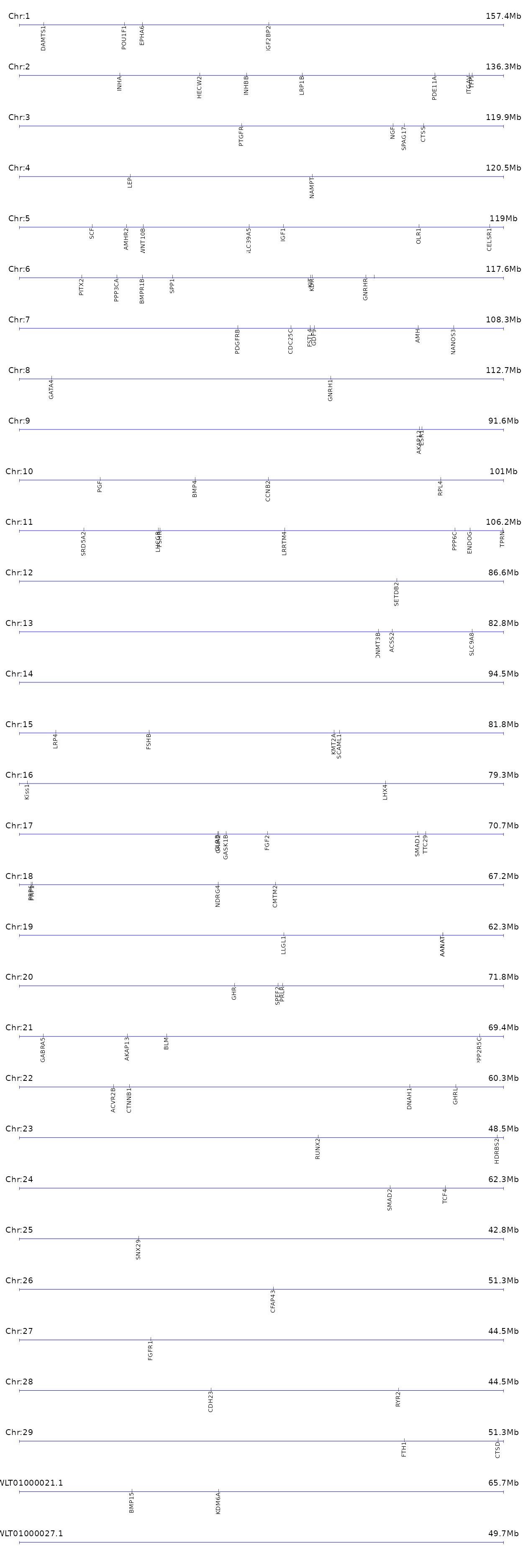

Supplement: Supplementary file 1 [file animals-15-00082-s001.zip › Supplementary Figure S1, chromosomal distribution.png]
